# Influence of Pea Protein Aggregates on the Structure and Stability of Pea Protein/Soybean Polysaccharide Complex Emulsions

**DOI:** 10.3390/molecules20035165

**Published:** 2015-03-20

**Authors:** Baoru Yin, Rujing Zhang, Ping Yao

**Affiliations:** State Key Laboratory of Molecular Engineering of Polymers, Department of Macromolecular Science, Fudan University, Shanghai 200433, China; E-Mails: 11110440024@fudan.edu.cn (B.Y.); 07300440001@fudan.edu.cn (R.Z.)

**Keywords:** aggregation, complex emulsion, homogenization, plant protein, soy polysaccharide, stability

## Abstract

The applications of plant proteins in the food and beverage industry have been hampered by their precipitation in acidic solution. In this study, pea protein isolate (PPI) with poor dispersibility in acidic solution was used to form complexes with soybean soluble polysaccharide (SSPS), and the effects of PPI aggregates on the structure and stability of PPI/SSPS complex emulsions were investigated. Under acidic conditions, high pressure homogenization disrupts the PPI aggregates and the electrostatic attraction between PPI and SSPS facilitates the formation of dispersible PPI/SSPS complexes. The PPI/SSPS complex emulsions prepared from the PPI containing aggregates prove to possess similar droplet structure and similar stability compared with the PPI/SSPS emulsions produced from the PPI in which the aggregates have been previously removed by centrifugation. The oil droplets are protected by PPI/SSPS complex interfacial films and SSPS surfaces. The emulsions show long-term stability against pH and NaCl concentration changes. This study demonstrates that PPI aggregates can also be used to produce stable complex emulsions, which may promote the applications of plant proteins in the food and beverage industry.

## 1. Introduction

Oil in water emulsions have attracted much attention in the fields of pharmaceutics, cosmetics, food, and beverage for encapsulation and protection of lipophilic bioactive components in aqueous phases [1,2,3] and also for increasing their oral bioavailability [4,5]. The use of plant proteins and polysaccharides as emulsifiers is of great interest to the food and beverage industry because of their nutritional properties, safety, and low cost [6,7]. Proteins possess good emulsifying capacity, but the emulsions stabilized by proteins are sensitive to environmental conditions, such as pH, ionic strength, and thermal processing [8,9]. Several polysaccharides, such as pectin, soybean polysaccharide, and gum Arabic, are naturally conjugated with hydrophobic proteins, and therefore can also be used as emulsifiers [10]. Compared with the emulsions produced from proteins, the emulsions produced from these polysaccharides have better stability against unfavorable environmental conditions because the carbohydrate part tends to stretch into the aqueous phase, thus preventing the droplets from aggregation and coalescence. However, polysaccharides display lower surface activity than proteins, hence leading to not only larger droplet sizes, but also a much higher polysaccharide concentration required to produce stable emulsions [11,12]. Consequently, protein-polysaccharide covalent conjugates and protein/polysaccharide electrostatic complexes have been exploited as emulsifiers in an attempt to take advantages of the properties of both the protein and polysaccharide. Compared with the production of protein-polysaccharide covalent conjugates, the preparation of protein/polysaccharide electrostatic complexes is much more convenient and time-saving [13,14]. Many protein/polysaccharide electrostatic complexes have been investigated for producing emulsions via a one-step method, *i.e.*, forming the protein/polysaccharide complex prior to emulsification [15].

Electrostatic attraction is essential for the formation of protein/polysaccharide electrostatic complex. Aside from chitosan, the other naturally charged polysaccharides are polyanions. The electrostatic complex of protein and anionic polysaccharide can form only in the pH range where the protein carries positive charges and the polysaccharide becomes deprotonated [16,17,18]. For the formation of plant protein/polysaccharide electrostatic complexes, the challenge is that such pH range is usually narrow and close to the isoelectric point (pI) of the plant protein, where both the solubility and emulsifying ability of the protein are poor [19,20,21]. To address this challenge, one approach is by first mixing the protein solution with a polysaccharide solution at higher pH, where the protein is soluble and carries negative charges, and then adjusting the mixture to an acidic pH to produce the complex. For example, Liu *et al.* prepared pea protein isolate (PPI) and gum Arabic (GA) complexes by mixing them together at pH 8.0 and then adjusting the mixture to the desired acidic pHs [19,21]. At a 2:1 PPI/GA ratio, soluble and insoluble complexes, formed at pH 4.23 and 3.77, respectively, could produce more stable emulsions compared with PPI alone [19]. Another approach is by respectively adjusting the protein and polysaccharide solutions to the pH where they carry opposite changes and then mixing them together. For example, we used acid soluble soybean protein, which contains more soluble protein in acidic solution, and soybean soluble polysaccharide (SSPS) to produce complexes in our previous work [22]. After the removal of large aggregates in the soybean protein solution by centrifugation at 6800 *g*, we mixed the soybean protein solution with the SSPS solution to produce dispersible complexes in the pH range of 3–4 where the protein and SSPS have opposite charges; the resultant complexes exhibited outstanding emulsifying ability. However, studies on the direct use of protein aggregates to produce stable protein/polysaccharide complex emulsions are limited to date.

Pea protein, extracted from pea seeds, possesses high nutrition and non-allergic properties as well as good functionalities [23]. Pea protein is mainly composed of 11S legumin and 7S vicilin and has a pI of about 4.3 [17]. Legumin is a hexameric protein constituted of polypeptides of about 60 kDa including an acidic subunit (about 40 kDa) and basic subunit (about 21 kDa) associated by disulfide bridges. Vicilin is a trimeric glycoprotein composed of polypeptides of about 50 kDa. Proteolysis of vicilin leads to smaller polypeptides: 33 kDa (αβ), 30 kDa (βγ), 19 kDa (α), 13.5 kDa (β), and 12.5–16 kDa (γ) [24]. Convicilin, a polypeptide of about 70 kDa, is considered as a third globulin pea protein [18]. Pea protein exhibits poorer solubility at pH around 5. Liu *et al*. reported that the solubility of PPI at pH around 5.5 was less than 30%, whereas the solubility increased to about 80% at pH values around 2.5 and 8 [19], indicating that the aggregation of pea protein at pH around pI is reversible.

SSPS, extracted from the byproduct of the isolation of soy protein [25,26], is composed of a main rhamnogalacturonan backbone branched with β-1,4-galactan, α-1,3- or α-1,5-arabinan chains and homogalacturonan which contains galacturonic acid (about 18% of total sugar). SSPS presents the advantages of high water solubility, high temperature stability, low bulk viscosity, and interfacial activity [27]. SSPS covalently links with a small fraction of hydrophobic protein which contributes to its interfacial activity [11,27,28]. SSPS carries negative charges at pH values above 3 where the carboxyl groups are deprotonated, therefore, SSPS can form complexes with positively charged proteins via both electrostatic and hydrophobic interactions [22,25,29]. 

**Scheme 1:**
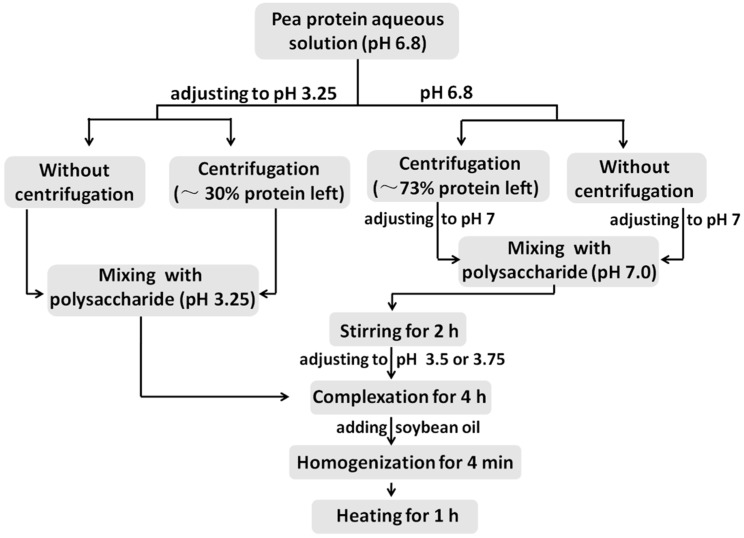
Flow chart of the four preparation approaches of PPI/SSPS complex emulsions.

Herein, we used the aggregates of PPI to form complexes with SSPS directly, and studied the oil-water interfacial activity and stability of the resultant complexes. In order to evaluate the effects of PPI aggregates, four approaches, which are illustrated in Scheme 1, were used to produce PPI/SSPS complex emulsions. These four approaches are as follows: (1) directly mixing PPI dispersion with SSPS solution at pH 3.25, where PPI and SSPS have opposite charges, and then emulsification, (2) mixing the supernatant of PPI dispersion with SSPS solution at pH 3.25 and then emulsification, (3) mixing the supernatant of PPI dispersion with SSPS solution at pH 7.0, where both PPI and SSPS carry negative charges, then adjusting the pH to 3.75 to produce electrostatic complex and emulsification, and (4) directly mixing PPI dispersion with SSPS solution at pH 7.0 then adjusting the pH to 3.5 and emulsification. The structure and long-term stability of the four resultant PPI/SSPS complex emulsions were carefully characterized. This study demonstrates that PPI aggregates can be utilized to produce stable PPI/SSPS complex emulsion.

## 2. Results and Discussion

### 2.1. Properties of PPI, SSPS, and PPI/SSPS Complex Solutions

**Figure 1:**
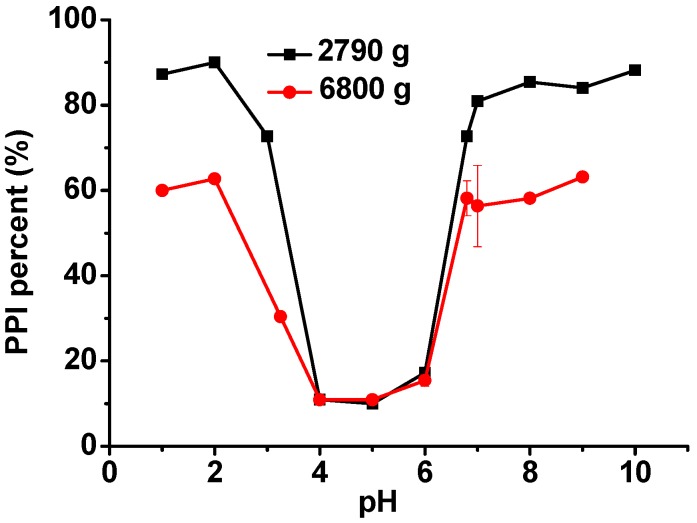
Remaining PPI percentages in different pH solutions after centrifugation at 2790 *g* or 6800 *g* for 30 min.

Firstly, the aggregation behaviors of PPI dispersed in different pH aqueous solutions were investigated. After centrifugation at pH 4, 5, and 6, only about 10% of the PPI remains in the solutions as shown in Figure 1, confirming that most of the PPI is insoluble at pH values around its pI. The PPI solubility increases when the solution pH is changed from 4 to 2 or from 6 to 10. At pH 2 and 10, about 89% of the PPI remains in the solution after 2790 *g* centrifugation, while only about 63% of the PPI remains after 6800 *g* centrifugation. This difference indicates that part of the PPI is still in aggregates, even when the solution pH is far away from its pI, but some of the aggregates are dispersible. In this study, the PPI used was a commercial one in which the aggregates possibly formed during the drying and storage process.

**Figure 2:**
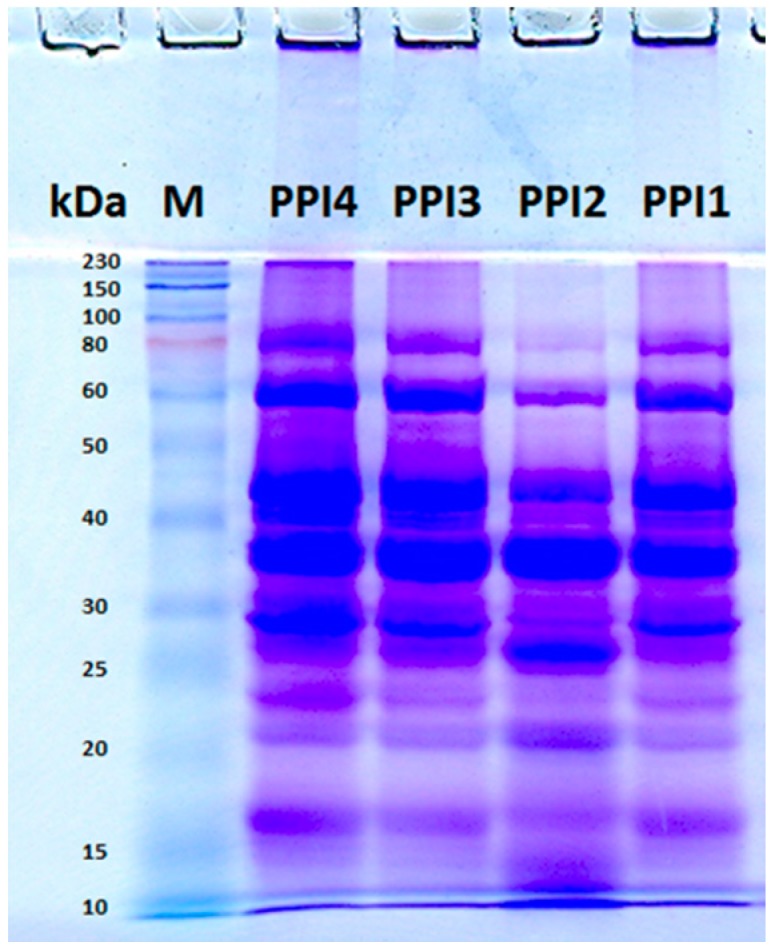
SDS-PAGE analysis of the four PPI solutions under reducing condition. Approximately 0.04 mg PPI was loaded in each lane.

Four PPI solutions without and with centrifugation at pH 3.25 and pH 6.8 were prepared as described in Table 1. The four PPI solutions were analyzed by sodium dodecyl sulfate polyacrylamide gel electrophoresis (SDS-PAGE) under reducing condition. The bands of the PPI samples shown in Figure 2 are similar to the bands reported in the literature [30]. PPI1 and PPI4 are the PPI samples without centrifugation; therefore, their bands are identical. PPI3 was obtained by 2790 *g* centrifugation at pH 6.8, whose bands are not significantly different from the bands of PPI1 and PPI4. PPI2 was obtained by 6800 *g* centrifugation at pH 3.25. Compared with the other PPI samples, PPI2 contains more components with smaller molecular weights and less components with larger molecular weights. This result indicates that the PPI components with larger molecular weights tend to aggregate at pH 3.25 and they can be removed by centrifugation.

**Table 1:** Preparation conditions of various PPI and SSPS stock solutions and PPI/SSPS complex solutions.

Sample	Preparation Condition	PPI Concentration (mg/mL)	SSPS Concentration (mg/mL)
SSPS	pH 7.0 or pH 3.25		50
PPI	pH 6.8	50	
PPI1	Adjusting PPI to pH 3.25		
PPI2	Centrifuging PPI1 at 6800 *g*		
PPI3	Centrifuging PPI at 2790 *g* and then adjusting pH to 7.0		
PPI4	Adjusting PPI to pH 7.0		
Complex1	Mixing PPI1 with SSPS at pH 3.25	5	25
Complex2	Mixing PPI2 with SSPS at pH 3.25	4	25
Complex3	Mixing PPI3 with SSPS at pH 7.0 and then adjusting pH to 3.75	5	20
Complex4	Mixing PPI4 with SSPS at pH 7.0 and then adjusting pH to 3.5	5	20

**Figure 3:**
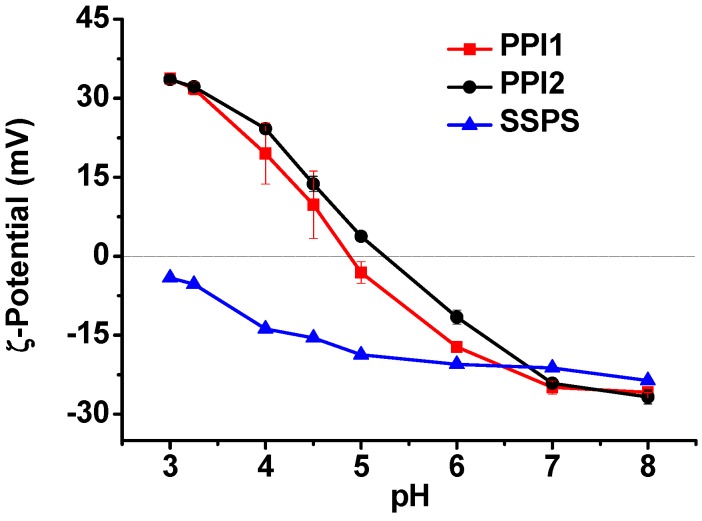
ζ-Potentials of PPI1, PPI2, and SSPS solutions as a function of pH.

Figure 3 shows the ζ-potentials of PPI1, PPI2, and SSPS in the pH range of 3–8. PPI tends to precipitate at pH values close to its pI. The ζ-potentials of PPI were measured immediately after magnetic stirring, therefore, the ζ-potentials are for reference only. PPI1 is positively charged at pH below 4.8 and negatively charged at pH above 4.8, while the pI of PPI2 shifts to pH 5.2. SSPS carries negative charges at pH above 3. The results in Figure 3 suggest that PPI1 and PPI2 can form electrostatic complexes with SSPS in the pH ranges of 3–4.8 and 3–5.2, respectively.

PPI, SSPS, and PPI/SSPS complex solutions prepared as described in Table 1 were investigated using dynamic light scattering (DLS). The PPI and SSPS concentrations in the solutions are shown in Table 2, which are the same as the concentrations used in emulsification process. The DLS results (Table 2) reveal that all the samples contain particles. PPI1 tends to precipitate as prepared; the corresponding particle size is about 1540 nm measured immediately after magnetic stirring. PPI2 has a size of about 206 nm because of the removal of larger aggregates by centrifugation. PPI4 shows a size of about 451 nm, substantiating that the aggregates produced at pH 7.0 are smaller than those produced at pH 3.25. SSPS also contains particles due to the aggregation of the naturally conjugated hydrophobic protein. Complex1 solution does not change significantly in appearance and in particle size compared with PPI1, suggesting that by directly mixing PPI aggregates with SSPS at pH where they have opposite charges cannot obtain dispersible PPI/SSPS complex. Complex2 solution displays larger particles than PPI2, but smaller particles than SSPS, suggesting the complexation of SSPS with PPI2. For Complex3 and Complex4 solutions, after respectively mixing PPI3 and PPI4 with SSPS at pH 7.0 and then adjusting the pH to 3.75 and 3.5, no precipitation was observed, confirming the formation of PPI3/SSPS and PPI4/SSPS complexes.

**Table 2:** DLS results of PPI, SSPS, and PPI/SSPS complex solutions.

Sample ^a^	PPI Concentration (mg/mL)	SSPS Concentration (mg/mL)	Without Homogenization		After Homogenization	
			D_h_^b^ (nm)	PDI ^c^	D_h_^b^ (nm)	PDI ^c^
SSPS	0	20	834 ± 53	1.0		
PPI1	5	0	1540 ± 276 ^d^	1.0		
PPI2	4	0	206 ± 8	0.60 ± 0.03		
PPI3	5	0	250 ± 75	0.59 ± 0.05		
PPI4	5	0	451 ± 66	1.0		
Complex1	5	25	1555 ± 455 ^d^	1.0	213 ± 9 ^e^	0.65 ± 0.04
Complex2	4	25	412 ± 18	0.48 ± 0.04	192 ± 4 ^e^	0.44 ± 0.03
Complex3	5	20	580 ± 42	0.36 ± 0.03	195 ± 8 ^e^	0.49 ± 0.03
Complex4	5	20	744 ± 88	0.57 ± 0.03	218 ± 9 ^e^	0.72 ± 0.02

^a^ The samples were measured directly without dilution. ^b^ Z-average hydrodynamic diameter. ^c^ Polydispersity index. ^d^ The measurement was performed immediately after magnetic stirring. ^e^ The measurement was performed after a pre-homogenization at 10,000 rpm for 1 min and then a homogenization at 850 bar for 4 min.

In this study, the emulsions were produced by high pressure homogenization, in which the oil droplets are disrupted by the forces of inertia and shearing in turbulent flow and also cavitation and shear stresses in laminar flow [31]. We also measured the PPI/SSPS complex solutions after the same high pressure homogenization as the emulsification process. The four complex solutions display similar particles of about 200 nm (Table 2), which are much smaller than the particles before homogenization. Furthermore, after homogenization, no precipitation was observed within at least one week, demonstrating that the high pressure homogenization disrupts the aggregates of PPI and SSPS and promotes the formation of dispersible PPI/SSPS complexes.

**Figure 4:**
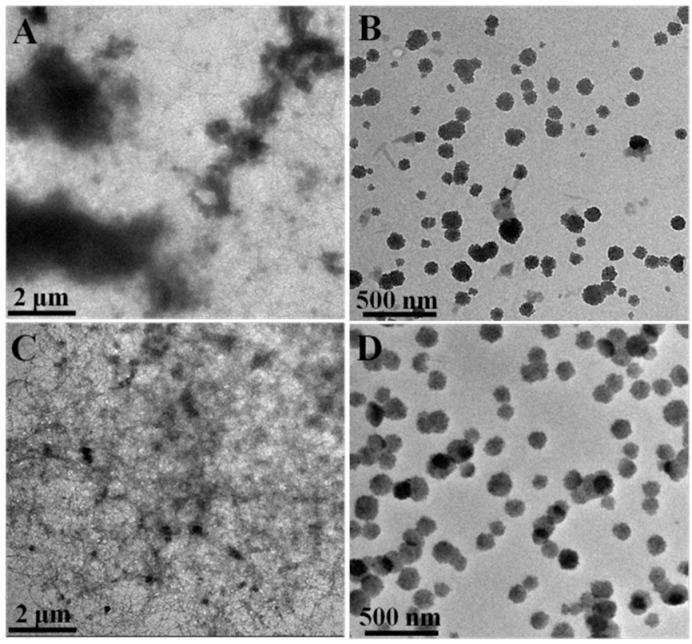
TEM images of Complex1 (**A**) before and (**B**) after high pressure homogenization as well as Complex2 (**C**) before and (**D**) after high pressure homogenization.

The morphologies of Complex1 and Complex2 before and after homogenization were observed by transmission electron microscopy (TEM). Before the homogenization, large aggregates with micron sizes exist in Complex1 sample (Figure 4A), whereas no large aggregates exist in Complex2 sample (Figure 4C) due to the centrifugation of PPI. After homogenization, both Complex1 and Complex2 samples present relatively uniform nanoparticles (Figure 4B,D). The TEM result is consistent with the DLS result shown in Table 2, supporting that the high pressure homogenization disrupts the aggregates of PPI and SSPS and promotes the formation of dispersible PPI/SSPS complexes.

**Figure 5:**
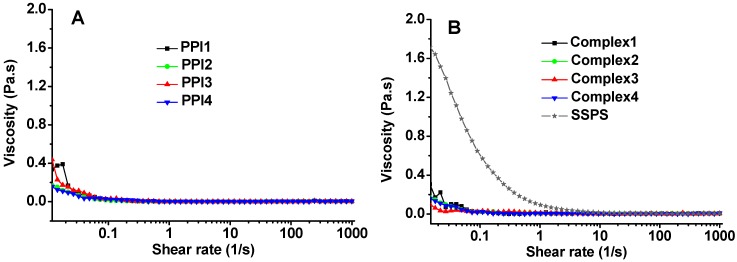
Viscosities of (**A**) PPI, and (**B**) SSPS and PPI/SSPS complex solutions as a function of shear rate.

The flow behaviors of PPI, SSPS, and PPI/SSPS complex solutions as a function of shear rate were investigated and the results are shown in Figure 5. The four PPI solutions exhibit similar low viscosity behaviors. The reasons may be as follows: (1) the PPI concentration is very low (only 4 mg/mL in PPI2 solution and 5 mg/mL in the other PPI solutions), (2) PPI is globular protein and/or in aggregation state, and (3) the inter-particle interactions are relatively weak which can be broken easily under low shear rates. When the shear rate applied is above 0.1 s^−1^, the viscosities of the four PPI solutions are almost the same as the viscosity of water. SSPS solution presents higher viscosity and shear-shinning behavior compared with the PPI solutions. However, the four complex solutions exhibit similarly low viscosity behaviors, distinctively different from the flow behavior of SSPS, indicating that the entanglement and hydrophobic interactions of SSPS have been broken in the complex solutions. Complex1 solution exhibits similar behavior to the other complex solutions, implying that PPI1 can also form complex with SSPS under shear stress despite the existence of large aggregates. The flow behaviors shown in Figure 5 further support the conclusion above that at acidic condition the PPI aggregates can form complexes with SSPS under shear stress.

PPI1 and PPI2 are positively charged in the pH ranges of 3–4.8 and 3–5.2, respectively, and SSPS is negatively charged when the pH is above 3 as shown in Figure 3. Therefore, electrostatic attraction between PPI and SSPS exists in the pH range of 3.25–3.75. In addition, SSPS contains about 6.3% hydrophobic protein, which can increase the interactions between PPI and SSPS. Before the high pressure homogenization, the self-aggregation of PPI blocks the interactions of PPI with SSPS and large aggregates still exist in Complex1 sample as shown in Table 2 and Figure 4A. The PPI aggregates can be disrupted by the shear stress or high pressure homogenization that promotes the interactions of PPI with SSPS. The formation of nano-sized PPI/SSPS complexes after the homogenization prevents the PPI from macroscopical precipitation. Table 2 and Figure 4 show that after high pressure homogenization, the PPI/SSPS complexes prepared from PPI with and without aggregates are almost the same; therefore, we can expect that the four complex emulsions produced by high pressure homogenization have similar structure and stability.

### 2.2. Emulsifying Ability of the Complexes and Stability of the Complex Emulsions 

PPI3 alone cannot produce stable emulsions within the pH range of 4–6 which is close to the pI of PPI. The PPI3 emulsions, produced at pH 3 and pH 7 with 5 mg/mL PPI in aqueous solution and 10 v% oil, have sizes of 273 and 398 nm, respectively. The resultant emulsions were adjusted to pH 2–8 and NaCl was added to reach 0.2 M concentration. After that, all the PPI3 emulsions display creaming within one week except for the emulsion produced at pH 3 without pH change and NaCl addition. PPI2 emulsion produced at pH 3 possesses a droplet size of 287 nm and presents similar creaming phenomena as PPI3 emulsion after the pH adjustment and addition of 0.2 M NaCl. Individual PPI1 and PPI4, which contain large aggregates, were also used as emulsifiers to produce emulsions at pH 3. After the homogenization, the PPI1 and PPI4 emulsions are not homogeneous. This result indicates that without SSPS, PPI aggregates alone cannot produce stable emulsion by high pressure homogenization. SSPS emulsion, produced at pH 3.75 with 20 mg/mL SSPS in aqueous solution and 10 v% oil, has much larger droplets than PPI emulsions, and creaming also appears within one week.

**Figure 6:**
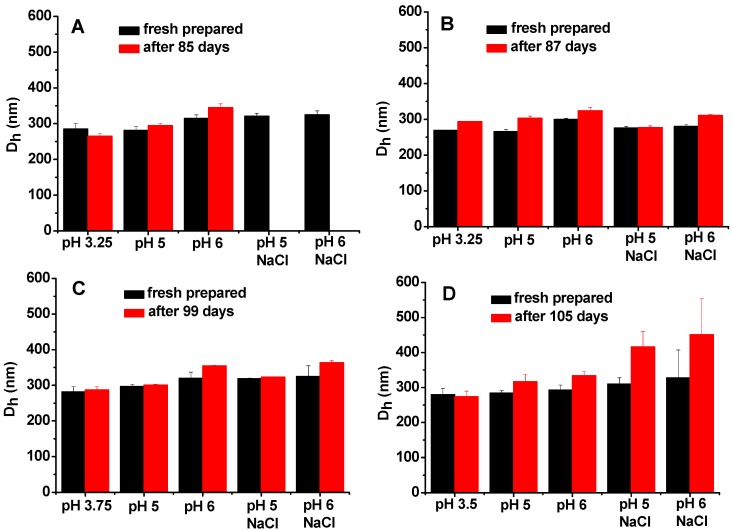
Droplet size changes of (**A**) Complex1, (**B**) Complex2, (**C**) Complex3, and (**D**) Complex4 emulsions before and after storage at 4 °C in different pH media with or without 0.2 M NaCl.

For Complex1 and Complex2, we chose pH 3.25 as the emulsification pH condition. The reasons are as follows: (1) in the pH range of 3–4.8, where PPI and SSPS carry opposite charges, the dispersible fraction of PPI at pH above 3.25 is too low as shown in Figure 1, and (2) the negative charges of SSPS at pH below 3.25 are too low as shown in Figure 3. Fresh prepared Complex1 and Complex2 emulsions, which have droplet sizes of 285 and 269 nm (Figure 6A,B), respectively, were adjusted to pH 5 and 6, and NaCl was added to reach 0.2 M concentration and then the emulsions were stored at 4 °C to investigate the long-term stability. After 85 days of storage, Complex1 emulsions stored at pH 3.25 are homogeneous, while a little whey layer at the bottom as well as increased droplet sizes are noted in the emulsions stored at pH 5 and 6 (Figure 6A), whereas the emulsions stored at pH 5 and 6 containing 0.2 M NaCl display creaming. Complex2 emulsions remain homogeneous after 87 days of storage at pH 5 and 6 with and without 0.2 M NaCl, suggesting that Complex2 emulsions are more stable than Complex1 emulsions. For Complex3 and Complex4, the optimal emulsification pH conditions are pH 3.75 and pH 3.5, respectively; the emulsions produced at other pH conditions have larger droplets after the pH change and NaCl addition (data not shown). Similarly, fresh prepared Complex3 and Complex4 emulsions with droplet sizes of 282 and 280 nm, respectively, were adjusted to pH 5 and 6, and the NaCl was added to investigate the stability. Complex3 and Complex4 emulsions are homogeneous after 99 and 105 days of the storage, respectively. On the other hand, after storage at pH 5 and 6 with 0.2 M NaCl, more significant changes in droplet size are noted in Complex4 emulsions compared with the changes in Complex 3 emulsions (Figure 6C,D).

**Table 3:** DLS results of PPI, SSPS, and complex emulsions before and after long-term storage in pH 6 with 0.2 M NaCl at 4 °C.

Sample		SDS Concentration after Dilution (%) ^a^	D_h_ (nm)	PDI
SSPS	fresh	0	724 ± 12	0.62 ± 0.08
	after 2 weeks	0	752 ± 23	1.0
		0.3	699 ± 17	0.27 ± 0.09
PPI1	cannot produce homogeneous emulsion			
PPI2	fresh	0	1094 ± 21	0.26 ± 0.05
	after 1 weeks	0	9025 ± 1008	1.0
		0.3	798 ± 17	0.77 ± 0.04
PPI3	fresh	0	1138 ± 15	0.13 ± 0.05
	after 2 weeks	0	8774 ± 198	1.0
		0.3	736 ± 28	0.81 ± 0.02
PPI4	cannot produce homogeneous emulsion			
Complex1	fresh	0	319 ± 8	0.27 ± 0.09
	after12 months	0	426 ± 26	0.14 ± 0.10
		0.3	371 ± 20	0.16 ± 0.03
Complex2	fresh	0	283 ± 7	0.16 ± 0.05
	after 14 months	0	331 ± 24	0.12 ± 0.02
		0.3	312 ± 12	0.16 ± 0.02
Complex3	fresh	0	325 ± 6	0.23 ± 0.06
	after 12 months	0	421 ± 28	0.25 ± 0.10
		0.3	388 ± 2	0.24 ± 0.13
Complex3 (unheated)	fresh	0	740 ± 70	0.74 ± 0.26
	after 12 months	0	3374 ± 245	1.0
		0.3	404 ± 50	0.60 ± 0.17
Complex4	fresh	0	328 ± 27	0.20 ± 0.03
	after 11 months	0	482 ± 178	0.31 ± 0.14
		0.3	404 ± 118	0.22 ± 0.09

^a^ The DLS samples were prepared by diluting 5 μL of the creaming layer with 3 mL of solution containing the same pH and NaCl concentration as well as 0 or 0.3% SDS.

After about 12 months of storage, all four complex emulsions stored in pH 6 medium containing 0.2 M NaCl display a creaming layer. In order to investigate the creaming mechanism during the storage, the creaming layer was analyzed using two methods: (1) the creaming layer was dispersed in water with the same pH and NaCl concentration followed by DLS measurement; (2) the creaming layer was dispersed in water with the same pH and NaCl concentration as well as 0.3% sodium dodecyl sulfate (SDS) followed by DLS measurement. SDS can disrupt the hydrophobic interactions and hydrogen bonding between droplets, thus can dissociate the aggregation/flocculation of the droplets. On the other hand, SDS cannot disrupt disulfide bonds, therefore, it cannot displace the protein and polysaccharide in the oil-water interface if disulfide bonds are involved in the interfacial film [32,33]. Table 3 shows the droplet sizes of the creaming layers after storage in pH 6 medium containing 0.2 M NaCl. For PPI2 and PPI3 emulsions, their droplet sizes increase to about 9 μm after short-term storage; however, by dispersing the creaming layer in 0.3% SDS solution, their droplet sizes drop to 798 nm and 736 nm, respectively. This result indicates that the creaming in PPI2 and PPI3 emulsions is mainly caused by the aggregation/flocculation of the droplets. Comparatively, for the four complex emulsions, their droplet sizes do not show significant difference when dispersing the creaming layers in the solutions with and without 0.3% SDS, indicating that the aggregation/flocculation and coalescence of the droplets are not severe after about one year of the storage. As mentioned above, all the emulsions display creaming layer after long-term storage in the media containing 0.2 M NaCl. Furthermore, Figure 6 shows that most of the samples increase their droplet sizes after the addition of NaCl. As we know, the density increases after the addition of NaCl in aqueous phase and NaCl can screen the electrostatic repulsion of the negatively charged droplets. Possibly, the increase of the density difference between the oil droplets and aqueous phase and the decrease of the electrostatic repulsion lead to the creaming of the complex emulsions in the media containing NaCl.

**Figure 7:**
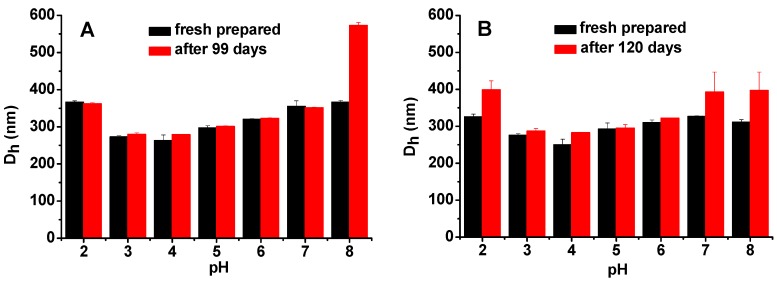
Droplet size changes of (**A**) Complex3 and (**B**) Complex4 emulsions before and after storage at 4 °C in different pH media without NaCl.

In this study, all the emulsions were heated at 90 °C for 1 h immediately after emulsification to produce an irreversible PPI/SSPS complex film at oil-water interface, which is similar to soy protein/SSPS emulsions reported previously [22]. During emulsification process, PPI and SSPS formed complex interfacial film because they have opposite charges as well as both PPI and SSPS have emulsification ability as shown in Table 3. After heating, PPI gelated [34,35] and thus part of the SSPS was embedded in the gelated PPI, which obstructs the dissociation of the complex film even when the PPI and SSPS both carry negative charges. This speculation is proved by the data shown in Figure 7. All the Complex3 and Complex4 emulsions after storage in the media of pH 2–8 for 99 and 120 days, respectively, are homogeneous. The droplet sizes increase from pH 4 to pH 8 and also from pH 4 to pH 2 due to the decrease of the electrostatic attraction and the increase of the electrostatic repulsion between PPI and SSPS (Figure 3). Figure 7 verifies that the heated complex emulsions are stable against pH change and long-term storage.

We also investigated Complex3 emulsion without heating for comparison. Table 3 shows that aggregation/flocculation occurs immediately after the fresh prepared emulsion being adjusted to pH 6. After 12 months of the storage at pH 6 with 0.2 M NaCl, the droplet size increases to about 3 μm, however, the size is only 404 nm when dispersing the creaming layer in SDS solution, demonstrating the aggregation/flocculation of the droplets during the storage. The droplet size change of the unheated Complex3 emulsion is similar to the change of PPI3 emulsion. Possibly, at pH 6, where both PPI and SSPS carry negative charges as shown in Figure 3, the electrostatic repulsion between PPI and SSPS drives hydrophilic SSPS to leave the droplet surface, which consequently results in the aggregation/flocculation of the droplets.

### 2.3. Structure and Morphology of the Complex Emulsions

**Figure 8:**
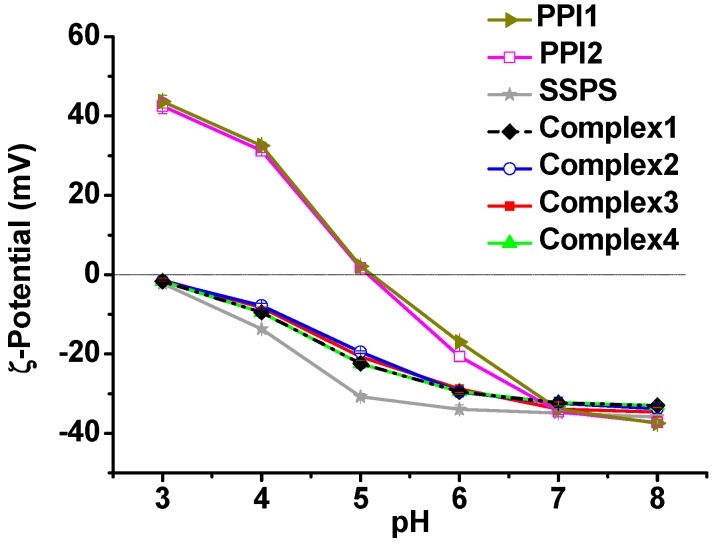
ζ-Potentials of PPI, SSPS, and complex emulsions as a function of pH.

Figure 8 shows the ζ-potentials of PPI, SSPS, and complex emulsions. The four complex emulsions display almost identical ζ-potential values at the same pH condition, suggesting that the four complex emulsions have similar surface structures. The ζ-potentials of the complex emulsions are different from those of PPI emulsions, and they are close to the ζ-potentials of SSPS emulsion, indicating that the droplet surfaces of the complex emulsions are covered by SSPS. Figure 8 shows that the complex emulsions are negatively charged in the pH range of 3–8. At pH 6, the ζ-potentials of the complex emulsions are about −28 mV. As discussed above, the electrostatic repulsion of the droplets can be screened by the addition of NaCl that may partly account for the creaming of the complex emulsions in the media containing 0.2 M NaCl.

**Figure 9:**
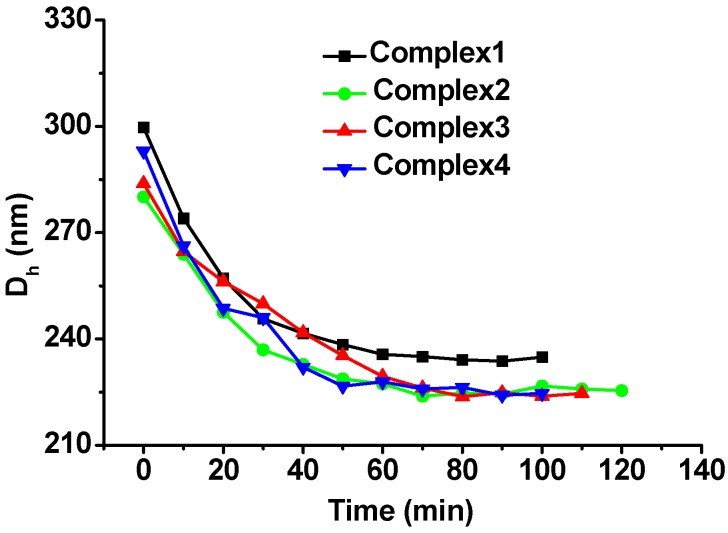
Droplet size changes of the four complex emulsions during the SSPS digestion by pectinase at pH 5 and 25 °C.

To further confirm the SSPS surfaces of the complex emulsions, pectinase was used to hydrolyze SSPS and the droplet sizes were measured in real time as reported previously [22]. Figure 9 shows that after addition of pectinase, the droplet sizes decrease gradually and then remain relatively constant. The thickness of SSPS layer is about 32 nm for Complex1, 27 nm for Complex2, 30 nm for Complex3, and 34 nm for Complex4, estimated from the decreases of their droplet sizes. This result demonstrates that the four complex emulsions have similar SSPS surfaces. Consequently, the four complex emulsions present similar long-term stability in the different media.

**Figure 10:**
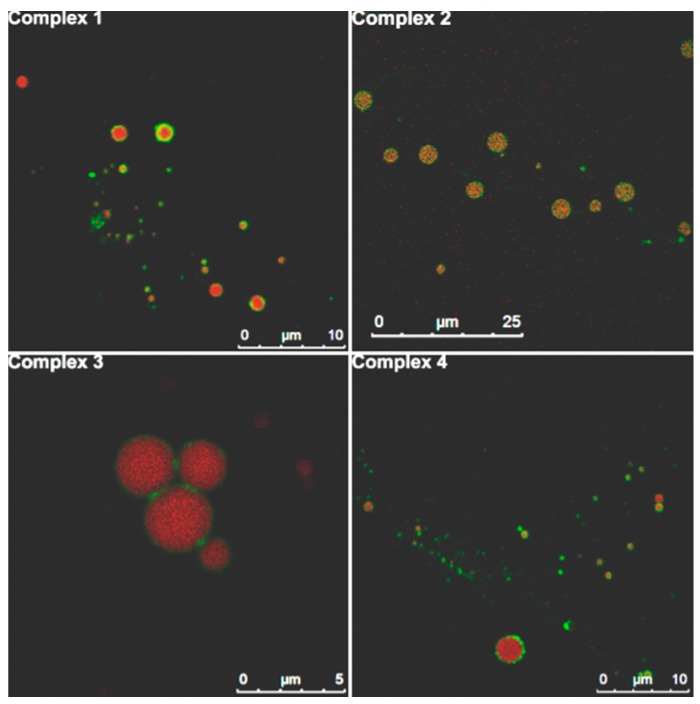
CLSM images of the oil droplet slices of the four complex emulsions produced from FITC-labeled PPI/SSPS complexes and 30 v% oil phase containing BPEN.

The droplets of the complex emulsions were observed on a confocal laser scanning microscope (CLSM) by labeling PPI with fluorescein isothiocyanate (FITC) and dissolving (9,10-bis(4-methoxyphenyl)-2-chloroanthracene (BPEN) in the oil phase. FITC emits green fluorescence and BPEN emits red fluorescence. Because CLSM can only observe the droplets with micron size, the emulsions were produced using 30% oil volume fraction to increase the droplet sizes. Figure 10 shows the slices from the middle of the dual fluorescence-labeled droplets. The four complex emulsions present similar microstructures: all the red fluorescent cores are enclosed by an integrated green fluorescent periphery. This result demonstrates that PPI locates at the oil-water interfaces in the four complex emulsions. Combining this result with the result that all the droplets of the four complex emulsions are covered by SSPS proved in Figure 8 and Figure 9, we can conclude that the four complex emulsions possess PPI/SSPS complex interfacial films.

**Figure 11:**
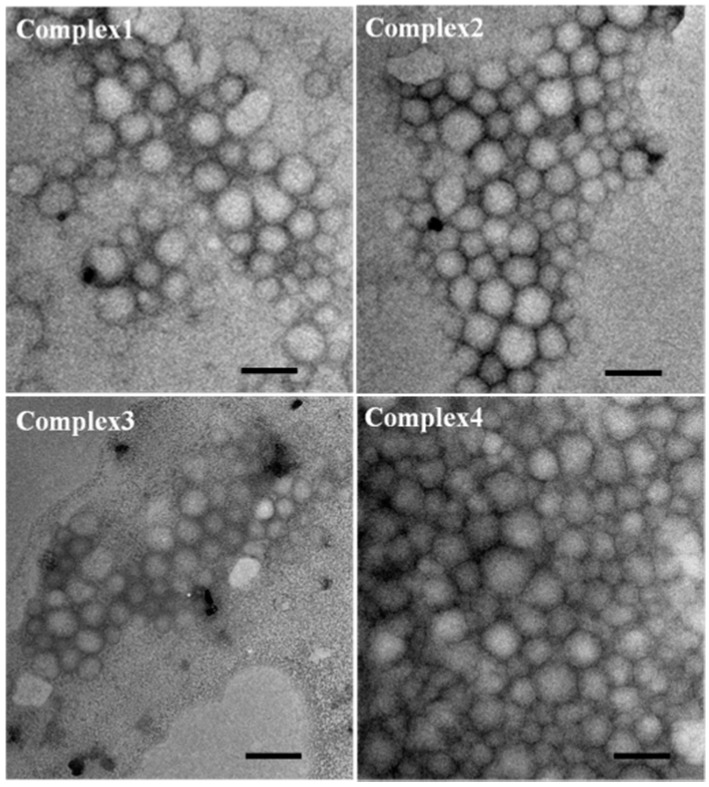
TEM images of the four complex emulsions prepared with 10 v% oil phase. The scale bars are 500 nm.

The droplets shown in Figure 10 are not homogeneous because a 30% oil volume fraction was used in order to increase the droplet sizes for better visualization under the microscope. For the four complex emulsions produced with 10% oil volume fraction, the TEM images in Figure 11 show that the droplets are homogeneous and the four complex emulsions do not present significant difference.

**Scheme 2:**
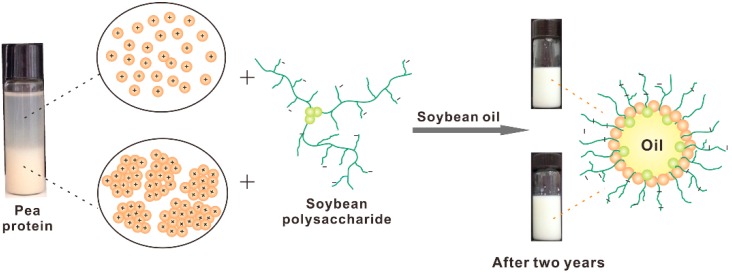
Illustration of the formation mechanism and structure of PPI/SSPS complex emulsions produced from PPI with and without aggregates as well as the photos of Complex2 (upper) and Complex1 (lower) emulsions after two years of storage.

Plant proteins have attracted much attention in the food industry [19]. However, their applications have been limited due to the acidic environment in most foods and beverages, in which plant proteins are not able to function effectively [25]. The dispersibility of PPI at the pH range around its pI is very poor, which is confirmed in Figure 1. This study reveals that the four PPI samples form similar complexes with SSPS at acidic pH under high pressure homogenization or shear stress (Table 2, Figure 4 and Figure 5). The four PPI/SSPS complex emulsions were produced by high pressure homogenization and heat treatment. Complex1 and Complex4 emulsions, produced from PPI containing aggregates, display similar droplet structure and similar stability as Complex2 and Complex3 emulsions produced from PPI in which the aggregates have been previously removed by centrifugation. Both complexation approaches, one is by mixing PPI with SSPS at pH 7.0 and then adjusting the pH to acidity, and the other is by mixing PPI with SSPS at acidic pH, can produce long-term stable complex emulsions. These results indicate that at acidic condition, high pressure homogenization disrupts the PPI aggregates and the electrostatic attraction between PPI and SSPS facilitates the formation of PPI/SSPS complex interfacial films. Scheme 2 illustrates the formation mechanism and structure of PPI/SSPS complex emulsions produced from PPI with and without aggregates as well as the photos of Complex1 and Complex2 emulsions after two years of storage. This study demonstrates that PPI without separation of the aggregates can also be utilized to produce stable complex emulsions, which may promote the applications of plant proteins in food and beverage industry.

## 3. Experimental Section

### 3.1. Materials 

Pea protein isolate (PPI; Pisane^®^ F9; dry matter 95.3%, protein 88.3%, fat ≤1.5%, carbohydrates ≤3.0%, and ash ≤6.0% based on dry matter) was from Cosucra Groupe Warcoing (Pecq, Belgium). Soybean soluble polysaccharide (SSPS; Soyafibe-S-CA100; crude protein 6.3%, moisture 5.6%, ash 7.4%) was from Fuji Oil Co., Ltd. (Osaka, Japan). Soybean oil and pectinase from *Aspergillus aculeatus* (≥3800 units/mL) were supplied by Sigma-Aldrich (Shanghai, China). Fluorescein isothiocyanate (FITC) and (9,10-bis(4-methoxyphenyl)-2-chloroanthracene (BPEN) were from Fluka. The other chemicals were analytical grade and from Sinopharm Chemical Reagent Co., Ltd. (Shanghai, China). All materials were used without further purification. All solutions were prepared using deionized water.

### 3.2. Solubility of PPI in Different pH Solutions

PPI was dissolved in water with an apparent concentration of 22 mg/mL. The solution was adjusted to desired pH values using 1 or 0.5 M NaOH or HCl solution. After equilibrium overnight, the PPI solutions were centrifuged at 2790 *g* or 6800 *g* for 30 min. The supernatants were lyophilized and the resultant powders were weighed to estimate the solubility of PPI in the different pH solutions.

### 3.3. Preparations of PPI and SSPS Stock Solutions and Four PPI/SSPS Complex Solutions

PPI stock solution was prepared by dissolving PPI in water with an apparent concentration of 50 mg/mL, and NaN_3_ with a final concentration of 0.02% was added to inhibit microbial growth. PPI1 stock solution was prepared by adjusting the PPI stock solution to pH 3.25 and then stirring the solution at 37 °C overnight. PPI2 stock solution was prepared by centrifugation of PPI1 stock solution at 6800 *g* for 30 min to remove the undissolved components; about 30% of the PPI remained in the solution after the centrifugation. PPI3 stock solution was prepared by stirring the PPI stock solution with pH 6.8 at 37 °C overnight and then centrifugation at 2790 *g* for 30 min and adjustment of the pH to 7.0; about 73% of the PPI remained in the solution after the centrifugation. PPI4 stock solution was obtained by stirring PPI stock solution with pH 6.8 at 37 °C overnight and then adjusting the pH to 7.0. SSPS stock solution with SSPS apparent concentration of 50 mg/mL and NaN_3_ concentration of 0.02% was prepared by dissolving SSPS and NaN_3_ in water, stirring the mixture at 37 °C overnight, and then adjusting the pH to 3.25 or 7.0. Complex1 and Complex2 were prepared by mixing PPI1 and PPI2 with SSPS at pH 3.25, respectively. Complex3 was prepared by mixing PPI3 with SSPS at pH 7.0 followed by 2 h stirring and then adjusting the mixture to pH 3.75. Complex4 was prepared by mixing PPI4 with SSPS at pH 7.0 followed by 2 h stirring and then adjusting the mixture to pH 3.5. The preparation conditions of the various PPI and SSPS stock solutions and PPI/SSPS complex solutions are summarized in Table 1.

### 3.4. Preparations of Four PPI/SSPS Complex Emulsions 

Each complex solution described above was stirred for 4 h then soybean oil was added to reach a volume fraction of 10%. The mixture was pre-emulsified using a homogenizer (FJ200-S, Shanghai Specimen Model Co., Shanghai, China) at 10,000 rpm for 1 min, and immediately afterward emulsified using a high pressure homogenizer (AH100D, ATS Engineering Inc., Shanghai, China) at 850 bar for 4 min followed by a heat treatment at 90 °C for 1 h. After overnight storage at 4 °C, the resultant emulsion was adjusted to desired pH values and 3 M NaCl solution was added to reach a final NaCl concentration of 0.2 M or not. The emulsions containing designed pH value and NaCl concentration were stored at 4 °C to investigate the stability.

### 3.5. Characterization 

SDS-PAGE under reducing condition was carried out on a gel electrophoresis apparatus (JM250, JM-X Scientific Co., Dalian, China) to analyze the components of the four PPI samples. Approximately 0.04 mg PPI was loaded in each lane. The gel was stained with Coomassie Brilliant Blue.

DLS measurements were performed on a laser light scattering instrument (Malvern Autosizer 4700, Malvern Instruments, Malvern, UK). The measurements were carried out at 25 °C and 90° scattering angle. The refractive indexes are 1.333 and 1.472 for water and soybean oil, respectively. The emulsions were diluted freshly before DLS measurement by adding 5 μL of the emulsion into 3 mL of aqueous solution containing the same pH and NaCl concentration.

ζ-Potentials were measured on a ZetaSizer Nano ZS90 (Malvern Instruments) at 25 °C. Before the measurement, PPI and complex solutions were freshly diluted to PPI concentration of 0.1 mg/mL with aqueous solution containing the same pH and 5 mM NaCl; similarly, SSPS solution was diluted to SSPS concentration of 1 mg/mL; PPI, SSPS, and complex emulsions were diluted by adding 7.5 μL of the emulsion into 3 mL of aqueous solution containing the same pH and 5 mM NaCl.

TEM visualizations were conducted on a FEI Tecnai G2 TWIN electron microscope (Portland, OR, USA). TEM samples were prepared by depositing a diluted complex solution or emulsion onto a carbon-coated copper grid and drying at room temperature.

Steady rheological measurements were conducted on a Bohlin Gemini 200HR-nano rotational rheometer (Malvern Instruments) at 25 °C with a cone-plate geometry of 40 mm diameter and 4° cone angle. The viscosities of the PPI, SSPS, and complex solutions were measured as a function of shear rate from 0.01 to 1000 s^−1^.

The SSPS on the droplet surface was hydrolyzed using pectinase. The hydrolysis was performed by adding 5 μL of original emulsion into 3 mL of pH 5.0 aqueous solution followed by adding 3 μL of 0.5% pectinase solution. The hydrolysis performed at 25 °C was monitored in real time by measuring the droplet size every 10 min.

The FITC- and BPEN-labeled emulsions were observed on a CLSM (Leica TSC SP5, Wetzlar, Germany). The excitation wavelengths for FITC and BPEN were 488 and 543 nm, respectively. FITC-labeled PPI was prepared at pH 9.5 as reported previously [22] with a weight ratio of FITC to PPI 1:200. FITC- and BPEN-labeled emulsions were prepared as described above except for using FITC-PPI and 30 v% oil containing 0.04% BPEN instead of PPI and 10 v% oil. The resultant emulsions were adjusted to pH 8.0 for observation.

## 4. Conclusions

The dispersibility of PPI at the pH range around its pI is very poor. In this study, we used PPI/SSPS electrostatic complexes as emulsifiers to evaluate the influence of PPI aggregates. By mixing PPI with SSPS at acidic pH as well as at neutral pH and then changing to acidic pH, Complex1 and Complex4 were produced from PPI containing aggregates, and Complex2 and Complex3 were produced from PPI in which the aggregates have been previously removed by centrifugation. The emulsions produced from the four complexes at acidic condition are long-term stable against the changes of pH and NaCl concentration. This study demonstrates that under acidic conditions, high pressure homogenization disrupts the PPI aggregates and the electrostatic attraction between PPI and SSPS facilitates the formation of PPI/SSPS complex films at oil-water interfaces. The conclusion that the aggregates of plant protein can also be utilized by complexation with polysaccharide to produce stable emulsions may largely broaden the applications of plant proteins in the food and beverage industry in the future.
